# Defining the “Cutoff” on the Urethral Caliber in Diagnosing a Female Urethral Stricture

**DOI:** 10.7759/cureus.57559

**Published:** 2024-04-03

**Authors:** Manu Kaushik Nagabhairava, Nikhil Khattar, Mahesh C Tripathi, Manasa T

**Affiliations:** 1 Department of Urology, M. S. Ramaiah Medical College, Bengaluru, IND; 2 Department of Urology, Medanta Medicity, New Delhi, IND; 3 Department of Urology, Shri Ram Murti Smarak (SRMS) Hospital, Bareilly, IND

**Keywords:** urethral caliber, urethral dilatation, female urethral stricture disease, female urethral stricture, female bladder outlet obstruction

## Abstract

Introduction

The rarity in detecting female urethral stricture (FUS) backed by the inconsistency regarding the cutoff on the caliber to direct any treatment for its increase poses a challenge to its existence. Therefore, the present study was conducted to determine the caliber of the urethra that clearly identifies a FUS.

Materials and method

In this prospective observational study conducted between November 2015 and July 2017, women with obstructive lower urinary tract symptoms (LUTS) and a history of relief on at least a single urethral dilatation were included if the American Urological Association (AUA) score was more than seven and the maximum flow rate (Qmax) was less than 20 mL/sec. Of the 71 women recruited, 10 women had recognizable external causes: caruncle (five), mucosal prolapse (three), and meatal stenosis (two). The remaining 61 underwent voiding cystourethrogram (VCUG) and urodynamics followed by urethrocystoscopy, if the findings suggested a stricture. A definitive diagnosis was sought in those without stricture disease. We categorized all patients as either having a “true” stricture or an alternate etiology. Categorical variables were presented in number and percentage (%) and continuous variables as mean ± standard deviation (SD).

Results

The mean dilatation ranged between one and six; the mean AUA score, ~17.82 ± 3.59; mean Qmax, ~10.21 ± 3.39 mL/sec; and the mean post-void residue (PVR), 106.65 ± 51 mL. A total of 29 patients were diagnosed to have stricture (dense = 17; flimsy = 12). None of the patients in this group had a urethral caliber of more than 14 French (Fr). Other etiologies were dysfunctional voiding (17), underactive bladder (seven), cystocele (four), and primary bladder neck obstruction (PBNO) (four).

Conclusion

Women with voiding LUTS should be screened for FUS only if the urethral caliber is ≤14 Fr.

## Introduction

The incidence of true urethral stricture in women with voiding urinary symptoms is less than 1% [1]. This lower incidence backed by the inconsistency regarding the cutoff on the caliber to direct any treatment for its increase poses a challenge to its existence. Powell et al. [2] have suggested that a 20 French (Fr) urethral caliber threshold is pathological, which was reiterated by Brannan [3] who advocated treatment in such cases. However, others have suggested that the 20 Fr caliber is too generous and have reduced the caliber to 16 Fr [4] or even up to 12 Fr [5]. The studies thereafter have relied on making a diagnosis of obstruction based on urodynamics, voiding imaging, and nomograms in addition to the use of the caliber. In a comprehensive systematic review regarding the outcomes of all surgical procedures for female urethral stricture (FUS), ranging from urethral dilatation to substitution urethroplasties, Osman et al. [6] recommended that a high-pressure low-flow pattern (urodynamic bladder outlet obstruction (BOO)) and the radiological imaging of a narrow segment should both be present along with a decreased urethral lumen (less than 20 Fr) to document FUS, leaving the controversy surrounding the cutoff yet to be addressed.

Therefore, we evaluated all women presenting to our department with a history of relief on previous urethral dilatation to determine the caliber of the urethra that clearly identifies a female urethral stricture. We chose this cohort as they were the population most likely to demonstrate a higher incidence of actual strictures in a smaller sample, given their lower incidence. In those women without a stricture, we attempted to define the alternative etiologies responsible for voiding lower urinary tract symptoms (LUTS).

## Materials and methods

In this prospective observational study conducted between November 2015 and July 2017, all women presenting to our outpatient department (OPD) with a history of voiding LUTS and symptomatic relief on urethral dilatation on any previous occasion were assessed. They were included, after obtaining informed consent, if they had an AUA symptom score of more than seven and a maximum flow rate (Qmax) of less than 20mL/sec on uroflowmetry. We excluded women with a history of neurogenic bladder dysfunction, prior bladder or urethral surgery, prior pelvic fracture/urethral disruption, and the concomitant presence of a genitourinary malignancy.

A detailed history followed by physical examination including meatal inspection, per vaginal examination, and focused neurological examination was performed. Urethral caliber assessment was done using Foley’s catheter beginning with 20 Fr and sequentially decreasing the size of the catheter by 2 Fr up to 10 Fr till the actual caliber was reached. If less than 10 Fr, an infant feeding tube of 9 Fr, 8 Fr, 7 Fr, 6 Fr, and 5 Fr were used in an analogous manner to assess the caliber. An ultrasound was done to look for upper tracts and the post-void residual urine. Those with obvious external causes such as caruncle, prolapse, and meatal stenosis were exempted from further investigation.

All patients underwent a voiding cystourethrogram (VCUG) and a urodynamic study (UDS) followed by urethrocystoscopy with a 13 Fr and 0° pediatric cystoscope to confirm the presence of stricture. Urodynamic BOO was defined by a high detrusor pressure (Pdet) (Qmax of more than 35 cm H2O) and low flow (Qmax less than 15 mL/sec) [7]. Stricture on VCUG was defined as narrowing in the middle or distal urethra with proximal urethral dilatation [6]. FUS was defined by a visible anatomical narrowing on urethrocystoscopy. Detrusor underactivity was defined by the presence of Pdet at Qmax less than 20 cm H2O, Qmax <15 mL/sec, and bladder voiding efficiency less than 90% [8]. Dysfunctional voiding required a combination of increased activity on electromyography (EMG) during sustained detrusor contraction on voiding phase in the absence of abdominal straining or failure to relax even in the absence of increased EMG activity during voiding [9]. Surface patch electrodes were used on either side of the anal opening during the UDS.

Statistical analysis

The data was recorded and analyzed using IBM SPSS Statistics for Windows, Version 22.0 (Released 2013; IBM Corp., Armonk, New York, United States). Categorical variables were presented in number and percentage (%) and continuous variables as mean ± standard deviation (SD). For the assessment of P value for the continuous variables, unpaired t-test was used. P values of less than 0.05 was considered as significant and indicated by "*" in the tables.

## Results

During our study period, a total of 82 female patients presented to our OPD with obstructive LUTS and a history of relief on prior urethral dilation. Eleven patients were excluded (prior history of pelvic fracture in two patients and bladder or urethral surgeries in nine patients: bladder augmentation in three patients, mid-urethral sling surgery in two, prior urethroplasty in two, and urethrovaginal fistula and urethral diverticulum in one patient each) from further evaluation. The final evaluation included 71 patients who satisfied the inclusion and exclusion criteria.

Demographic criteria and baseline characteristics are indicated in Table 1.

**Table 1:** Demographic and baseline characteristics of the study population

Variable	Mean ± standard deviation (n = 71)
Age (years)	47.15 ± 10.1
No of prior dilatations	2.85 ± 1.18
AUA	17.82 ± 3.59
Peak flow rate (Qmax) (mL/sec)	10.21 ± 3.39
Post-void residual urine (mL)	106.65 ± 51.1

About 88.7% of the cases presented with predominantly voiding LUTS (poor flow with incomplete voiding), followed by a recurrent acute urinary retention (AUR) in 11.3% of patients. Upper tracts were normal in all patients with a mean serum creatinine of 0.93. External causes for voiding LUTS were noted in 10 patients on local examination which included caruncle (five), urethral mucosal prolapse (three), and meatal stenosis (two). They were not subjected to further investigations.

In the remaining 61 patients, VCUG, UDS, and urethroscopy were performed. Urethral caliber was assessed in all patients at the time of UDS. Of these, 29 patients were finally diagnosed of a stricture urethra on urethroscopy. The characteristics of these patients is shown in Table 2. None of the patients in this group had a urethral caliber of more than 14 Fr. UDS in all these patients was clearly suggestive of BOO with a high pressure and low flow. VCUG was suggestive of stricture (dilated proximal urethra and convexity of urethral outline) in 28 cases. On urethroscopy, 12 patients had a flimsy stricture which, though visible clearly as a narrowing with flimsy membranes, easily broke on advancing the cystoscope sheath, whereas the remaining 17 had a dense fibrotic narrowing which gave stiff resistance to the urethroscope. Four patients with stricture had a component of dysfunctional voiding on EMG, three of them had flimsy strictures of calibers (14 Fr in two and 12 Fr in one), whereas one had a dense stricture with a caliber of 6 Fr.

**Table 2:** Comparison of findings in patients with and without stricture AUA: American Urological Association; PFR: peak flow rate; PVR: post-void residue

Variables	Stricture (n = 29)	Nonstricture (n = 32)	P values
AUA symptom score	17.59 ± 4.03	17.91 ± 3.23	0.7324
Number of dilatations	2.93 ± 1.14	2.87 ± 1.29	0.8589
Serum creatinine (mg/dL)	0.91 ± 0.18	0.91 ± 0.28	0.9754
PFR (mL/sec)	8.23 ± 3.22	11.32 ± 2.59	0.0001
PVR (mL)	113.20 ± 61.02	98.37 ± 49.77	0.3006
Caliber (Fr)	9.21 ± 2.43	15.25 ± 1.98	<0.0001

Four more patients had an anatomical obstruction in the form of cystocele. These women had no stricture on urethroscopy although two of them had urethral calibers of 14 and 12 Fr and the one with 12 Fr caliber also showed urodynamic BOO. The rest of the 28 patients did not have a stricture (confirmed by urethrocystoscopy in all patients). These characteristics are shown in Table 2.

Although all patients had a free uroflowmetry Qmax of less than 15 mL/sec, urodynamic BOO was present in only six of these (four of them were finally diagnosed of primary bladder neck obstruction (PBNO), and two had dysfunctional voiding who also showed the area of stricture like narrowing on VCUG). Eleven of these patients had a urethral caliber of ≤14 Fr (PBNO in two patients, hypocontractile detrusor in one patient, and dysfunctional voiding in eight patients). 

Dysfunctional voiding was present in 17 patients (mean International Prostate Symptom Score (IPSS) of 18.2, mean Qmax of 10.1 mL/sec, mean post-void residue (PVR) of 107.5 mL). The urethral caliber was 14 Fr or less in eight of these patients (14 Fr in six, 12 Fr in one, and 10 Fr in one). Proximal urethral ballooning on VCUG was seen in four of these patients. Urodynamic BOO was present in five patients.

Underactive detrusor was present in seven patients (mean IPSS of 18.2, flow rate of 10.2 mL/sec, mean PVR of 95.22 mL). The urethral caliber was 12 Fr (<14 Fr) in one patient. In addition to poor contraction, one patient had poor compliance in the filling phase, and one patient had detrusor hyperactivity with impaired contractility (DHIC).

PBNO was present in four patients (mean peak flow rate (PFR) of 9.7 mL/sec and mean PVR of 114 mL, VCUG showing closed bladder neck with increased PVR, UDS demonstrating BOO, and cystoscopy excluding anatomic narrowing).

Overall, 29 of the 42 patients with a urethral caliber of ≤14 Fr had stricture, while none of the patients with a caliber of more than 14 Fr had stricture. A total of 28 of the 35 patients, who had a proximal urethral dilatation on VCUG, harbored a stricture, and all had urodynamic BOO (four had dysfunctional voiding, and three had underactive detrusor). In 30 patients with both proximal dilatation and urodynamic BOO, 28 had stricture, and two patients had dysfunctional voiding (calibers 10 Fr and 18 Fr).

## Discussion

There have been few studies to specifically measure normal and abnormal female urethra [10]. The normal mean adult female urethral caliber in Westerners is 22 Fr (range 18-28 Fr) [11]. Hole [12] observed the caliber to be between 18 and 26 Fr and that the caliber of women with and without urethral syndrome is not significantly different. It is not very different in Asian women as observed by Chang et al. [13]. Though various studies have included a cutoff above which they did not do a urethroplasty, none have specifically studied to define a cutoff between a strictured and nonstrictured female urethra. Osman et al. [6] stated that most of these studies used a cutoff of ≤14 Fr along with a urodynamic BOO. In our study, we found that nothing short of a cystoscopic demonstration of a narrowing could define a FUS.

All our patients with FUS had a caliber of less than 14 Fr. On the contrary, 13 patients with a urethral caliber of less than 14 Fr did not have a stricture (dysfunctional voiding in eight patients, cystocele and PBNO in two patients, each, and underactive detrusor in one patient). Two of these showed a proximal urethral dilatation on VCUG, and one patient even had a urodynamic BOO. Dysfunctional voiding was the most common diagnosis to give a false impression of FUS on calibration while demonstrating a VCUG and urodynamic picture identical to stricture. This could be the plausible reason why the urethra appears tight, and hence, patients are inadvertently subjected to urethral dilatation. Interestingly, three of the patients from the stricture group had features of dysfunctional voiding on EMG, and all of them had flimsy strictures. It is possible that the stricture in these women was a result of unwarranted repeated urethral dilatation, as suggested by Smith et al. [14].

Functional causes for obstruction are far more common in women than a stricture. Dysfunctional voiding and PBNO are recognized as the chief functional causes of BOO in neurologically intact women [15]. Dysfunctional voiding is defined by the International Continence Society as an intermittent and/or fluctuating flow rate due to intermittent contractions of the peri-urethral striated muscles during a sustained voluntary detrusor contraction in a neurologically normal woman. This was diagnosed using a combination of increased EMG activity during voiding in the absence of abdominal straining or radiographic evidence of contraction of the sphincter or failure of the sphincter to relax even in the absence of increased EMG activity during voiding [16]. Kuo [7], in his videourodynamic categorization of 207 women with BOO, identified obstruction at the level of the sphincter and at the level of the pelvic floor as separate entities with an incidence of 27.1% and 57.2%, respectively, apart from PBNO which was seen in 8.7% of patients (FUS accounted for only 6.8% in that series). Kuo recently coauthored one of the largest experiences of evaluation and categorization of 1914 women with LUTS using videourodynamics and reported on 810 women with BOO in the series [16]. Only 6% of cases had anatomical obstruction; out of which; stricture was identified in only 3.7% cases. They further subcategorized patients with dysfunctional voiding to "dysfunctional voiding proper" in 325 patients (40.1%), which was dyssynergic external sphincter at the mid-urethra and "poor relaxation of the external sphincter" (PRES) in 337 patients (41.6%), which is at the level of the pelvic floor in the distal urethra. In dysfunctional voiding proper, the proximal urethra is dilated till the mid-urethra level on video, and the intermittent increase in the EMG activity of the striated muscle causes an increase in the voiding pressure, whereas in PRES, the narrowing is visible in the distal part of the urethra, and the EMG activity during voiding phase may not increase but just does not fall silent as in normal voiding, giving rise to poor flows with not so increased voiding pressures. Sinha, in a review on dysfunctional voiding, argued that only the needle electrode EMG can specifically diagnose peri-urethral striated muscle activity, whereas surface electrodes pick the activity which is a combination of the sphincter muscle as well as the pelvic floor muscle [17]. 

Despite the absence of stricture, women did experience a relief on urethral dilatation (UD). As observed by Basu et al. [18], UD does have temporary benefit in symptoms in women with an overactive bladder, voiding dysfunction, and all BOO which is not proven to be a FUS. The same has been confirmed urodynamically with a significant improvement in Pdet at Qmax at six months post-dilatation [19].

BOO in females is relatively uncommon with a reported incidence of 2.7%-8% in those presenting with lower urinary tract symptoms [1], and the incidence of female urethral stricture in those with BOO is 4%-13% [7]. Such a low incidence should prompt the urologist or the treating physician to identify other causes of LUTS rather than subjecting the female to unnecessary urethral dilatation for LUTS, which itself can be a cause for stricture [14] and is of questionable value.

Once a routine practice for all “urethral syndrome,” office-based urethral dilatation has all evidences piled up against it, but the practice refuses to die. In a study from Texas in 1999, Lemack et al. [20] observed that 61% of the 642 urologists offer urethral dilatation for urethral syndrome, and the trends to offer were significantly lower in those urologists trained within that decade as compared to those trained earlier. A decade later, in 2008, Santucci et al. [21] came up with the concern of this ongoing practice of office dilatation in women for nonspecific conditions such as recurrent infections, overactive bladder, interstitial cystitis, or atrophic vaginitis and the enormous economic impact of this practice on the healthcare system as recorded from the National Health Statistics records from the United States. The authors speculate poor dissemination of the scientific evidence to the practicing urologist as one of the main reasons for the prevalent practice. From our study, we observe that none of the women were being dilated for recurrent urinary tract infections, interstitial cystitis, or any arbitrary urinary symptoms, but all of them had severe voiding LUTS as is evident from their mean Qmax and mean IPSS which were not different in strictured and nonstrictured groups. This points more toward lack of clarity in protocols to follow in female voiding LUTS in the available literature and guidelines.

Our patients were a highly selected group who not only had obstructive symptoms but were also getting symptomatically relieved on urethral dilatations. The caliber of the urethra in all these patients was less than 20 Fr (mean ≈9.2 Fr). Still only 40.85% had true stricture. The cutoff that we used, i.e., ≤14 FR, also failed to conclusively diagnose stricture in 13/42 patients (30.95%) which could be confirmed only after subjecting them to urethrocystoscopy. This confirms the suggestions made in the only meta-analysis on FUS where FUS was defined as “a symptomatic, anatomical narrowing of the urethra based on a failure of catheterization, urethral calibration, visual inspection, or endoscopy or radiography” and not simply a cutoff value of the urethral caliber [6]. They further concluded that for the diagnosis of FUS, a caliber of less than 20 Fr with urodynamic obstruction pattern and a radiological depiction of strictured segment were mandatory [6]. None of our 19 patients who had a caliber between 15 and 18 Fr were finally diagnosed of having FUS. Our findings are just short of concluding that women should be evaluated by a urethroscopy for FUS only if the urethral caliber is ≤14 Fr. However, since ours is a highly selected group of patients who were on dilatation, the caliber cutoff may have been different when compared to previous studies.

The cutoff did not define stricture. Out of 42 women with a caliber of ≤14 Fr, only 69% had stricture. The simultaneous presence of urodynamic BOO or proximal urethral dilatation can also be misleading, and only a stricture confirmed on urethroscopy should be subjected to treatment directed toward it.

Limitations

These findings may not be applicable for all women with obstructive LUTS, and further studies with larger cohorts are required to define the exact cutoff of the caliber for urethral stricture.

## Conclusions

Our study concludes that women with voiding LUTS, even if they experienced improvement with previous urethral dilatation and have a urodynamic BOO along with imaging suggestive of urethral narrowing, should be screened for FUS by a urethroscopy only if the urethral caliber is ≤14 Fr.
